# Perceptions of fentanyl among African Americans who misuse opioids: implications for risk reduction

**DOI:** 10.1186/s12954-023-00915-6

**Published:** 2023-12-19

**Authors:** Khary K. Rigg, Ethan S. Kusiak

**Affiliations:** Department of Mental Health Law and Policy, University of South Florida, 13301 Bruce B. Downs Blvd, Tampa, FL 33612 USA

**Keywords:** Fentanyl, African Americans, Overdose deaths, Opioid misuse, Heroin

## Abstract

**Background:**

Fentanyl is a powerful synthetic opioid that is 50 times more powerful than heroin and has become ubiquitous in the illicit drug supply in the USA. Studies show that among people who use drugs, fentanyl is sometimes viewed as a desirable substance due to its high potency and low cost, while others have an unfavorable perception because of its association with overdose. Unfortunately, studies on the perceptions of fentanyl are relatively rare and existing studies tend to rely on samples with little African American representation. The objective of this study, therefore, was to identify and describe perceptions of fentanyl among African Americans who misuse opioids, while capturing their motivations for seeking out or avoiding fentanyl.

**Methods:**

In-depth interviews (*n* = 30) were conducted with a sample of African American adults who misuse opioids in Southwest Florida between August 2021 and February 2022. Audiotapes of interviews were transcribed, coded, and thematically analyzed.

**Results:**

Analyses revealed the presence of three subtypes of fentanyl-related perceptions: (1) fentanyl as an avoided adulterant, (2) fentanyl as a tolerated adulterant, and (3) fentanyl as a drug of choice.

**Conclusions:**

These findings show that African Americans’ perceptions of fentanyl are not monolithic and suggest the distribution of fentanyl test strips and naloxone may be an effective risk reduction strategy. Given that most studies on fentanyl rely on quantitative data from drug seizures and death certificates, this study uniquely contributes to the literature by capturing the voices of African Americans who use drugs.

## Introduction

Opioid overdose deaths continue to be a serious public health problem in the USA. In 2021, 80,411 Americans died of an opioid overdose, the most ever recorded in a calendar year [1]. It is important to note, however, that the type of opioid causing the most deaths has changed over the years. In fact, the opioid overdose crisis is often divided into distinct waves based on spikes in deaths due to different types of opioids [2]. The first wave began in 1999 with a dramatic rise in deaths due to prescription opioid pills [3]. The second wave began in 2010, with spikes in overdose deaths involving heroin [4]. The third wave began in 2013, with significant rises in deaths due to illicitly manufactured fentanyl (and its many analogues) [5]. Fentanyl remains the opioid type responsible for the most deaths in the USA, accounting for more fatalities than prescription opioids and heroin combined [4].

In the last decade, fentanyl-related deaths in the USA have risen at an exponential rate, doubling almost every three years since 2013 (3105 deaths in 2013 to 36,359 deaths in 2021) [1, 6]. These spikes are a result of increased fentanyl production and trafficking [7]. Fentanyl is easier and cheaper to produce than heroin. Heroin requires dealers to grow large crops of poppy plants which can be spoiled by bad weather/harvest, whereas fentanyl’s ingredients are synthetic, easier and cheaper to acquire, and less susceptible to poor environmental conditions [8, 9]. Additionally, fentanyl is up to 50 times more potent than heroin, which means smaller amounts translate into larger profits for dealers [10, 11]. Fentanyl has become so ubiquitous in the street drug supply that it is not only being put into heroin and counterfeit opioid pills, but also showing up in supplies of marijuana, cocaine, and methamphetamine [12–14].

In addition to its low production costs and high potency, fentanyl also has a rapid onset of action [15]. Fentanyl’s rapid onset increases risk of death because life-threatening respiratory depression can occur within two minutes after injection, compared to 10 min for heroin [15, 16]. Additionally, fentanyl’s psychoactive effects have been known to fade more quickly than heroin [17]. The shorter duration of a fentanyl high results in people having to inject more frequently, which also exacerbates overdose risk. Despite its ubiquity and lethality, relatively few studies have examined risk perceptions of fentanyl among people who use drugs and the existing literature is somewhat mixed.

On the one hand, studies have shown that fentanyl is sometimes viewed as an undesirable drug due to its association with overdose and death, leading persons to engage in avoidance strategies (e.g., returning/discarding drugs believed to be laced with fentanyl) [10, 18]. Similarly, studies have also shown that fentanyl is sometimes perceived negatively because of its undesirable physiological side effects (e.g., shortness of breath, blurry vision) and a shorter lasting high [10]. On the other hand, not all persons who use drugs view fentanyl negatively. Research shows that some may intentionally seek out fentanyl because of perceived advantages over heroin or prescription opioid pills [19]. Some individuals have a favorable view of fentanyl due to its higher potency, which can be attractive to those with higher opioid tolerance [20]. And for individuals in communities where lower-purity heroin is the norm, fentanyl’s stronger potency can be particularly appealing [18, 21].

Understanding perceptions of fentanyl is important because such perceptions can influence how people use the drug and if/which protective measures are taken to avoid overdose. There is also reason to believe that African Americans may have unique risk perceptions of fentanyl as previous studies have documented racial differences in perceived risks of certain drugs, including opioids [22–24]. Unfortunately, studies on the perceptions of fentanyl are relatively rare and existing studies tend to rely on samples with poor racial diversity or do not stratify findings based on race. As a result, little is known regarding how African Americans view fentanyl and we do not know the extent to which findings, such as those previously stated, generalize to African Americans. Studies that focus specifically on African Americans are especially timely because they now have one of the highest rates of fentanyl overdose deaths in the USA [25]. In fact, a recent report showed that more African Americans died from fentanyl overdoses than any other drug in 2021 and at far higher rates than Whites or Hispanics [26]. According to the report, African Americans died from fentanyl overdoses at more than twice the rate of Hispanics and a rate 27% higher than that of Whites.

### Current study

This study provides data on how fentanyl is viewed in an understudied population that has high rates of fentanyl deaths. To our knowledge, this is the first study to examine this issue specifically among African Americans who misuse opioids. A better understanding of African Americans’ perceptions of fentanyl could help identify intervention opportunities to address fentanyl-related overdose deaths in this population. The objective of this study was to identify and describe perceptions of fentanyl, while capturing their motivations for seeking out or avoiding fentanyl. This study provides a unique contribution to the literature in the utilization of a qualitative methodology. As such, this study has the potential to shed new light on fentanyl use among African Americans through a more contextual understanding of their lived experience.

## Methods

### Sample

The data in this article are drawn from the Florida Minority Health Study, a research study on African American opioid misuse funded by the University of South Florida through a seed grant program. Data collection was conducted from August 2021 to February 2022 throughout the Tampa Bay area, a metropolitan region of southwest Florida. Thirty participants were recruited for the qualitative component of the Florida Minority Health Study. Only persons 18 years old or over who identified as African American and reported past 90-day opioid misuse were eligible to participate. Opioid misuse was defined as: (1) any use of heroin or illicit fentanyl, and (2) use of a prescription opioid (e.g., oxycodone, hydrocodone) without a prescription or in a manner not prescribed by a doctor. All study protocols and instruments were reviewed and approved by University of South Florida’s Institutional Review Board.

### Recruitment

Purposive sampling strategies were used to recruit participants, including posting flyers in predominantly African American neighborhoods, at bus stops, in community health centers, and on social media (Twitter and Facebook). Additionally, numerous local drug treatment centers throughout southwest Florida agreed to post our flyers on their bulletin boards and keep study cards in their lobby/waiting area. Chain referral was also used, whereby each participant who completed an interview could refer others whom they thought might be eligible. Participants were not paid for referrals. Recruitment materials explained that the Florida Minority Health Study was a study about the health and substance use of African Americans and interested persons were instructed to go to a website for eligibility screening.

### Data collection and procedures

Once eligibility was determined, participants were provided (online) an informed consent form to read and sign. Participants were assured that their participation was strictly confidential, and they could stop participating at any time. After the consent form was electronically signed, participants were asked to complete an online Qualtrics survey including demographic (e.g., age, income, educational attainment, gender), psychosocial (e.g., mental health diagnoses, housing status), and substance use (e.g., alcohol, marijuana, cocaine, heroin) variables. This survey took about 20 min to complete. Following the survey, participants took part in an individual interview to capture more detailed information on their opioid use patterns. A total of 30 participants were interviewed. Participants were paid $40 (via Amazon gift card) for completing the survey and another $40 (via Amazon gift card) for completing the individual interview. Interviews typically lasted about 40 min and were conducted by the first author using Microsoft Teams.

Individual, semi-structured, in-depth interviews were conducted as a directed conversation, allowing each interview to take its own course and unfold naturally [27]. Participants were asked a series of open-ended questions that explored their perceptions of fentanyl, motivations for seeking out/avoiding fentanyl, and strategies for mitigating risk of fentanyl overdose. Examples of the questions on fentanyl included: (1) What are your thoughts about fentanyl? (2) Have you ever knowingly/accidentally taken fentanyl? (3) Why do you seek out/avoid fentanyl? (5) How concerned are you that the drugs you take might have fentanyl in it? (6) What steps do you take (if any) to protect yourself from fentanyl overdose? These questions were selected on the basis that they were clearly worded, open-ended, and conversational in nature. Overall, the goal was for the interview to resemble a casual conversation where the participant does the majority of the talking.

### Data analysis

Each interview was audio recorded in Microsoft Teams using the record feature. The audio file was then transcribed verbatim using Otter.ai software and then evaluated for accuracy by the research team. Transcripts were then imported into NVivo, a qualitative data analysis software program [28]. The interview transcripts were analyzed by the second author using thematic analysis [29, 30]. This is a widely used method for reporting patterns and themes within interview data. It was chosen because it is a flexible technique and is appropriate in cases such as this, where the research topic is understudied. Additionally, thematic analysis is particularly useful when the research goal is to richly describe important themes relating to a phenomenon of interest. The following paragraph briefly summarizes Braun & Clarke’s six-step process for conducting thematic analysis [29].

Phase one involved becoming familiar with the data by reading each transcript twice. Initial ideas for coding were noted on the second reading in the memo feature of the software program (similar to writing observations in the margins). The second phase is where initial codes were generated. Perceptions and behaviors related to fentanyl were systematically coded across the entire data set. Once all data were initially coded and collated, step three began. This involved sorting the codes into potential themes and gathering all the text data relevant to each initial theme. Phase four consisted of reviewing and refining the devised set of initial themes by checking if the data cohered together meaningfully within each theme. Phase five is where the specifics of each theme were decided upon and the overall story of the data emerged, generating clear definitions and names for each theme. In the sixth and final phase, the report was written and compelling excerpts from participants were chosen to illustrate each theme. After 30 interviews, it was determined that saturation was achieved as no new patterns were emerging from the data. The themes related to fentanyl perceptions are further discussed in the following sections.

## Results

### Sample characteristics

Demographic characteristics for the sample are displayed in Table 1. The total sample (*n* = 30) included 14 males and 16 females, ranging in age from 18 to 46 (mean = 35 years; median = 37 years). All participants identified as African American. Two-thirds of the sample (66.6%) had a high school diploma or less, while only 4% had a bachelor’s degree. Forty-three percent were unemployed and approximately 97% made less than $40,000 in annual income. Regarding living arrangement, half reported homelessness or living in public housing, while the other half rented or owned their own home. Over two-thirds (70%) reported a history of arrest. Mental health problems were also common, with 60% reporting depression and 56.6% reporting anxiety. Participants reported use of a range of substances. Regarding past year use, heroin (86.7%), alcohol (76.7%), prescription opioids (74.3%), and marijuana (66.7%) were the most common. The following section describes themes related to how fentanyl was viewed across the sample.

**Table 1:** Sample characteristics

Measure	Total (*n* = 30)
Age	*n* (%)
18–25	4 (13.3)
26–34	10 (33.3)
35–44	13 (43.3)
45+	3 (10.0)
Gender	
Male	14 (46.7)
Education attainment	
Some high school	8 (26.6)
High school diploma/GED	12 (40.0)
Some college	6 (20.0)
Completed a bachelor’s degree	4 (13.3)
Employment status	
Employed full-time	8 (26.6)
Employed part-time	9 (30.0)
Unemployed	13 (43.3)
Total personal income (past year)	
$0–$9999	8 (26.7)
$10,000–$19,999	5 (16.7)
$20,000–$29,999	10 (33.3)
$30,000–$39,999	6 (20.0)
$40,000+	1 (3.3)
Health insurance	
Medicaid	10 (33.3)
Affordable Care Act	5 (16.7)
Private health insurance	3 (10.0)
Uninsured	12 (40.0)
Living arrangement	
Pay rent for housing	12 (40.0)
Homeowner	3 (10.0)
Public housing	11 (36.7)
Homeless	4 (13.3)
History of arrest	
Arrested	21 (70.0)
Depression diagnosis	
Yes	18 (60.0)
Anxiety diagnosis	
Yes	17 (56.6)
Drug use (past year)	
Alcohol	23 (76.7)
Marijuana	20 (66.7)
Crack cocaine	9 (30.0)
Powder cocaine	13 (43.3)
Heroin	26 (86.7)
Rx opioid misuse	22 (73.3)

### Fentanyl as an avoided adulterant

Fentanyl was most commonly seen as an undesirable adulterant of heroin (50% of sample) to be avoided. There were several reasons for fentanyl being viewed as undesirable. The first and most prominent reason was because of its close association with overdose and death. Stories of friends either being sent to the hospital or losing their lives because of fentanyl were prevalent among participants in this category. It was also common for participants to report personally overdosing on fentanyl (unintentionally) at least once in their lives. Frequent reports of fentanyl deaths in the news and on social media also contributed to fentanyl’s lethal reputation as an adulterant to avoid. Risk of overdose was the primary motivation for avoiding fentanyl, as articulated by the following participant:

“If you haven’t heard about fentanyl, you’re living under a rock! I don’t fuck with it because it’s a killer. I wanna get high, not die. I avoid it like the plague. Too many people I know died from that shit. I don’t want nothing to do with it. If it goes left, I go right. If it goes right, I’m going left. It’s too dangerous to take a chance. I’m gonna get ‘clean’ one day, but people who fuck with fentanyl won’t live to see that day. I will.” (35 year old male).

Fentanyl was also undesirable because it was perceived to have an inferior high compared to heroin. Participants often described the fentanyl rush as being “too strong,” not allowing them to gradually enjoy or savor the experience. These quick, intense highs were not described in favorable terms and participants complained that fentanyl too often “knocked them out” or made them “nod off” too quickly. In addition to fentanyl being overly potent, the quality and length of the high was viewed as lacking. Fentanyl highs were described as being “too short,” as well as “murkier” or “dirtier” than heroin. This participant explains why the psychoactive and physiological effects caused her to avoid fentanyl as much as possible:

“Oh, you can tell when it’s fentanyl. It hits different. Not different good, different bad. It reminds me of Special K, like an animal tranquilizer that makes you disassociate. It’s a dirty feeling. And before you know it, you’re knocked out because it’s so strong. There’s no gradual high. Instead of feeling high, you go straight into ‘nodding out’ if you manage not to overdose…It also makes you sick, you feel like shit the next day.” (28 year old female).

Even though participants believed fentanyl was being cut into the majority of heroin, they still engaged in several avoidance or risk reduction strategies. In an attempt to detect fentanyl, some would try to smell or look for its presence in their heroin. It was believed that fentanyl was darker in color and that a browner shade of heroin was a strong indication of adulteration. Fentanyl was also thought to have a distinct smell, with some claiming that it can give off a “sugar” or “ammonia” scent. In cases where significant fentanyl adulteration was suspected, participants reported using heroin in smaller amounts initially, and then gradually increasing the amount to avoid overdose. This participant explains how he attempted to detect fentanyl in his heroin and the risk reduction strategies he engaged in:

“The color will tell you. Down here (in Florida), brown heroin is more a mixture of everything (adulterated), and white means more pure. Any time we’d get white heroin, we knew it was pure. It’s the opposite up north. Anytime we’d get brown heroin up north, we knew it was good…Whenever we had a batch with bad color, we did a little to see how it was, and then we would adjust the next hit. But if the color was ok, we’d dive in.” (37 year old male).

### Fentanyl as a tolerated adulterant

For some (23.3% of sample), fentanyl was not viewed as a feared adulterant to be avoided, but rather as a new “ingredient” that adds to the variety of available heroin. The prevailing sentiment was that due to the ubiquity of fentanyl in the heroin supply, avoiding fentanyl entirely was all but impossible. Fentanyl was not feared, but rather tolerated. Instead of fear, fentanyl was described in a neutral or matter of fact manner. The realization that fentanyl was “here to stay” and cut into so much of the product being sold as heroin made participants feel that actively trying to avoid it was futile. Participants in this category already assumed fentanyl was in their heroin and “acted accordingly,” as this individual articulates:

“Fentanyl doesn’t freak me out. There are worst things in life than fentanyl. I mean, it’s the new normal. It’s everywhere. People might as well get used to it. It is what it is. You can’t buy heroin anymore without fentanyl in it. I assume every bag (of heroin) has fentanyl and act accordingly. Running from it is a waste of time. That’s the reality of our situation.” (37 year old male).

Because it was viewed neutrally, fentanyl was neither sought out or avoided. Participants acknowledged the ubiquity of fentanyl in the heroin supply, but it was not foremost on their minds. The possible presence of fentanyl also did not significantly factor into if or how they used heroin. Other factors such as cost, availability of injection equipment, and avoiding law enforcement featured more prominently in their decision making about heroin use. This participant explains how much she thinks about fentanyl when she is using heroin:

“*I’m not stressing about fentanyl. I know it’s out there. But if I’m being honest with you, I’m not checking my shit for fentanyl before I shoot it, fuck that! Who has time for that? Even if I find out it (heroin) has other stuff in there, I’m still gonna use it. I’m not throwing it away, I’ll get dope sick…I get that fentanyl is dangerous, but it’s not the boogeyman like everyone makes it out to be. I already know it’s in there.*” *(41 year old female)*.

### Fentanyl as a drug of choice

Fentanyl was also viewed by some as their drug of choice (26.7% of sample). Participants in this category were unique in that they viewed fentanyl as a standalone drug and not simply an adulterant. In fact, fentanyl was seen as a preferred option to all other opioids. Compared to fentanyl, opioid pills were expensive and difficult to acquire, and heroin tended to be weaker and cost more. For these participants, opioid pills and heroin were only used in situations that fentanyl could not be acquired. Rather than negative or neutral, fentanyl was viewed favorably by participants and discussed in positive terms. This participant explains how he views fentanyl:

“Once I tried it (fentanyl), I was like, ‘oh this is really strong.’ Why am I running from this? It’s cheaper and I only need a little. What’s not to like? Once I switched over (from heroin to fentanyl), I never looked back. Fentanyl is easier to get these days, so it’s easier for me to find…I don’t see it (fentanyl) as this super dangerous thing…The only time I’ll use heroin is when I can’t get my hands on any (fentanyl).” (35 year old male).

Fentanyl was viewed as the drug of choice for several reasons. First, fentanyl was preferred because of its strength and was often described as getting the most “bang for your buck.” Longtime users of fentanyl would often claim their tolerance was so high it no longer made economical sense to use heroin due to having to purchase so much to get high. Another reason fentanyl was favored over other opioids was the lower cost. Fentanyl tended to be cheaper compared to heroin and opioid pills, which was an appealing bonus especially given fentanyl’s higher potency. Higher availability also played a role in making fentanyl their drug of choice. Participants claimed that dealers were less likely to run out of fentanyl than heroin or opioid pills. Some even claimed that it was not uncommon to come across dealers who only sold fentanyl. This participant discusses the main reasons for fentanyl being his preferred option:

“Why do I prefer fentanyl? First of all, it’s cheaper and its everywhere! You can’t argue with cheap. Second of all, its stronger and my body has gotten used to it. Whenever I use heroin now, I have to use a shit ton to not get sick. It ends up costing me too much…With heroin, you don’t know what’s in that shit. With fentanyl, at least I know what I’m taking so I don’t die.” (30 year old male).

Another reason for their preference of fentanyl was the ability to use fentanyl intranasally and still get high. Using intranasally was a way for some to avoid “track marks” and reduce risk of overdose. Not having to inject also removed the need to walk around with injection equipment (e.g., needles, syringes) which lowered the likelihood of being caught by police with drug paraphernalia. Some also claimed using intranasally meant they were less likely to contract HIV or develop skin infections. The following participant explains why she decided to start using fentanyl intranasally:

“I realized I couldn’t go back (to heroin) because my tolerance was sky high. I started thinking, ‘okay if I’m gonna be using this shit (fentanyl) every day, I gotta be safe about it.’ That’s when me and my friends started snorting it, too. Snorting heroin don’t get you all that high, but I can snort this (fentanyl) and it still gets you high. This way (snorting), I can stay safe and still get high…I still shoot (fentanyl), but not as much as I used to.” (28 year old female).

## Discussion

The results of this study indicate the presence of three subtypes of fentanyl perceptions. First, we found that the majority of participants viewed fentanyl as a dangerous adulterant that should be actively avoided. Fentanyl was viewed as undesirable not only due to the heightened risk of overdose, but also because it produced a shorter and inferior quality high. This is consistent with previous research that also documented similar complaints about fentanyl among people who use drugs [18]. For example, a Massachusetts-based study found that one reason fentanyl was disliked was because of unpleasant side effects and shorter intoxication that required more frequent administration [10]. Our findings contribute to the literature by documenting that some African Americans who use drugs have a considerable fear of fentanyl and actively try to avoid it.

What was also concerning were the avoidance/risk reduction strategies that were reported. It was believed that fentanyl could be detected by smell or simply by looking at the color of heroin. This belief that fentanyl can be detected via smell and color has also been found in other studies [10]. Fentanyl, however, is odorless and there is no evidence that it gives off a distinct scent. Additionally, illicit fentanyl comes in many forms and does not have a specific color. Fentanyl can appear white in some cases, but it may also look off-white, tan, or even brown [31]. Our findings show that some individuals are utilizing smell and color to detect fentanyl and then use this information to make decisions on whether/how to use their heroin. This is concerning because these decisions are likely being made using unreliable information. Risk reduction interventions should focus on correcting these myths about fentanyl and educating users that fentanyl can only be reliably detected with chemical detection tools, such as fentanyl test strips [32]. This finding also supports the need for wider distribution of fentanyl test strips for pre-consumption drug checking and naloxone (known as the opioid overdose antidote), particularly among African Americans, as it appears that attempts are being made to detect fentanyl using unreliable methods.

Second, we also found that fentanyl was viewed as a tolerated adulterant. These participants viewed fentanyl in a more neutral way (as opposed to negative) and did not actively engage in avoidance strategies. The presence of fentanyl did not prominently factor into their decision making about if/how to use heroin. This finding was troubling in that it shows some individuals are engaging in opioid misuse without giving much thought to the presence of fentanyl in their drugs. Without a clear appreciation for fentanyl’s lethality, individuals may use in ways that are likely to result in overdose (e.g., use too much). Given this finding, risk reduction interventions might include public health messaging that increases knowledge about the high prevalence of fentanyl in the illicit drug supply. We also suggest messaging that focuses on raising awareness that fentanyl can also be found in counterfeit opioid pills purchased from dealers as this was not widely known among participants.

Third, we found that fentanyl was also viewed as a drug of choice by some participants. These individuals actively sought out fentanyl, using it on a daily basis, only using heroin or opioid pills when fentanyl was unavailable. While this finding was alarming, individuals seeking out fentanyl are not entirely new [19]. Previous studies have documented that fentanyl is increasingly being sought out for a variety of reasons [18, 21]. Some studies have found that fentanyl is sought out for its potency and intense rush [20, 21]. We found that in addition to these reasons, fentanyl was also preferred because it was cheaper, more available, and allowed for intranasal use that still resulted in a strong high. Our findings build on previous research by showing that not only is fentanyl viewed favorably by some African Americans, but it is also actively sought out in some cases for its perceived advantages over other kinds of opioids. Given that these individuals routinely use fentanyl, a substance much stronger than heroin or opioid pills, overdose risk may be unique and elevated among this group. Such individuals should always carry naloxone and educate their loved ones that more than one dose may be necessary to effectively reverse a fentanyl overdose.

Our findings should be interpreted within the context of the study limitations. As this was a Florida-based non-probability sample, generalizations should be made with this in mind. Because individual interviews were used as a means of data collection, social desirability and interviewer bias may have been a possibility. Additionally, interviews were done remotely on Teams, rather than in-person, which may have influenced the type of responses. However, these effects are believed to have been mitigated through the use of an experienced interviewer who was also African American. The inherent limitations of self-report data (e.g., recall bias) also apply to this research. Additionally, multiple coders were not used which could increase the possibility of discovery failure. Future research should investigate the extent to which these results can be duplicated in rural parts of the USA and internationally, where drug cost and availability may differ. Studies should also attempt to quantify the prevalence of each subtype among African Americans who misuse opioids and assess the extent to which harm reduction strategies would be acceptable among this population.

These limitations notwithstanding, the data presented here are especially important as African Americans now have one of the highest fentanyl death rates in the USA [33]. Although the overdose crisis has been studied extensively over the last two decades [34, 35], to the author’s knowledge, this is the first study to explore perceptions of fentanyl among African Americans and document that these perceptions are not homogenous. This research used qualitative methods of data collection and analysis to gain a fuller understanding of how fentanyl is perceived by African Americans and their motivations for seeking out or avoiding fentanyl. Ultimately, our findings suggest that tailored harm reduction strategies are needed for individuals with different perceptions of fentanyl. Given that most studies on fentanyl rely on quantitative data from drug seizures and death certificates [7, 36], this study uniquely contributes to the literature by capturing the voices of African Americans who use drugs.

## Data Availability

The datasets used and/or analyzed during the current study are available from the corresponding author on reasonable request.
